# Tests with proven value in diagnosis of COVID-19

**Published:** 2020-06

**Authors:** Zahid Hussain Khan, Shahram Samadi, Jalil Makarem, Seyed Mohamad Mireskandari

**Affiliations:** Department of Anesthesiology and Intensive Care, Imam Khomeini Hospital Complex, School of Medicine, Tehran University of Medical Sciences, Tehran, Iran

**Keywords:** COVID-19, Diagnostic tests, Lockdown

## Abstract

COVID-19 has literally ravaged the entire world. People from all walks of life are badly affected because of compulsory lockdown around the world. Timely diagnosis is a problem as there is no single test that can achieve the highest acceptable sensitivity. Some of the tests are indeed costly and footing the bill by the governments can cause a tremendous load on the Treasury. As it stands, the current tests are beyond patient means and, thus, the patient would never have it performed. Lastly, there is no consensus as to whether everyone should be tested for COVID-19 and not based on presence of clinical features. Unfortunately, since the disease has been declared a pandemic, all should be considered to be infected unless provenother-wise by the tests that are performed.

Ever since the coronavirus disease (COVID-19) emerged for the first time on December 2019 in China, its correct diagnosis and its therapeutic management has remained a bone of contention for the intelligentsia and academia. Different tests are currently available such as the polymerase chain reaction (PCR), imaging and many others but there is hardly any consensus as to when exactly should these tests be performed in those infected or else those who are potentially vulnerable as they live in endemic or to be more exact pandemic areas. Thus, the common man feels panicked and in total oblivion as to whom to refer to and which test would be needed so that they take a sigh of relief that they are Corona free and are doing well.

In their elegant study, Chanet al. (1) showed that the novel COVID-19 RdRp/Hel assay exhibited the highest sensitivity (Se) and specificity (Sp) for detection of SARS-CoV-2 RNA *in vitro* and in COVID-19 patient specimens. Compared to the RdRp-P2 assay, it was found to be statistically more significant in both respiratory tract and non-respiratory tract clinical specimens. This method currently identifies those tested negative by the RdRp-P2 assay in nasopharyngeal aspirate/swab or throat swab, saliva and plasma specimens.

The COVID-19 RdRp/Hel assay for testing saliva instead of the nasopharyngeal aspirates and sputum is a landmark achievement (2) because nasopharyngeal aspirates are seemingly difficult to access because one has to follow the trajectory medial to the middle turbinate of the nose to reach the area above which is not only cumbersome but requires an added skill and expertise besides inflicting a significant degree of stress and may also trigger a forceful cough or sneeze thus spreading infection.

Real-time reverse transcriptase polymerase chain reaction (rRT-PCR) of nasopharyngeal swabs has been used to confirm the clinical diagnosis (3). At times chest CT-Scan is used as an important compliment to the rRT-PCR test for the diagnosis of COVID-19 because it has a higher Se reaching up to 97% (4). The question arises as to why the chest CT-Scan should not be used as a primary tool for detection of COVID-19 in epidemic areas where the patients show a greater vulnerability to infection when it has been found and unequivocally stated that chest CT-Scan could reveal pulmonary abnormalities consistent with COVID-19 in patients with initial negative rRT-PCR results (5).

Apparently, if chest CT-Scans are routinely taken in epidemic zones in patients with clinical symptoms, it would definitely lead to a tremendous financial burden on the Treasury, but at the same time would result in instantaneous diagnosis of the disease by the classical appearance of ground-glass opacities, consolidation and interstitial findings thus helping the clinician in clinching the final diagnosis of COVID-19 and initiating timely and immediate treatment before the lungs are rendered ineffective and the patients end up in intensive care units under controlled mechanical ventilation with a high mortality rate.

Other than the chest CT-Scan, the currently available diagnostic tests for the coronavirus disease have a low positive predictive value for the disease because of a low Se and moderate Sp. Thus, we are witnessing a large number of false positives or else would be missing too many true positives. Although false positive has been cited as a challenge for the CT-Scan, nevertheless it can be accepted in its face value as none of the patients would be missed. However, false negative values with this test are almost negligible which is its added advantage as very few would be declared as Corona free. Statistically it has been argued and universally accepted that tests that have low or nil false negative values fare well as no patient with evolving symptoms of COVID-19 would be missed, or in simple words all vulnerable patients would receive due surveillance which is the legal right of all the patients. However, as chest CT Scans incur tremendous expenses, it should preferably be used as a complimentary test with the other prevailing molecular tests if the diagnosis is in doubt and not unequivocal.

Casting a critical look at the available tests and tools and their potential costs in diagnosis of COVID-19, and the fact that many patients might be from the less affluent parts of the world, the question that arises in one’s mind is which of the tests should be advocated to achieve the fastest and greatest dividends without inflicting a lethal blow to an individual’s shattered economic status because of the compulsory prevailing lockdown and a total extinction of jobs for the bread winners of families.

Mounesan et al. (6) in their comprehensive article emphasize that countries should further focus on strengthening their diagnostic capacities, community participation, and the utilization of the hard-learned lessons learned in the affected countries, particularly those with identical socio-economic conditions.

## Responsibility and ethical requirements.

Neither the submitted manuscript nor another copy with substantially similar content under our authorship has been published in any language or is being considered for publication elsewhere. We take responsibility for the integrity of the work as a whole, from inception to the published article.
